# Aquatain® Mosquito Formulation (AMF) for the control of immature *Anopheles gambiae sensu stricto* and *Anopheles arabiensis*: dose-responses, persistence and sub-lethal effects

**DOI:** 10.1186/1756-3305-7-438

**Published:** 2014-09-16

**Authors:** Oscar Mbare, Steven W Lindsay, Ulrike Fillinger

**Affiliations:** International Centre of Insect Physiology and Ecology (icipe) -Thomas Odhiambo Campus, Mbita, 40305 Kenya; Disease Control Department, London School of Hygiene & Tropical Medicine, London, WC1E 7HT United Kingdom; School of Biological and Biomedical Sciences, Durham University, Durham, DH1 3LE United Kingdom

**Keywords:** *Anopheles gambiae sensu stricto*, *Anopheles arabiensis*, Vector control, Surface film, Larval source management

## Abstract

**Background:**

Persistent monomolecular surface films could benefit larval source management for malaria control by reducing programme costs and managing insecticide resistance. This study evaluated the efficacy of the silicone-based surface film, Aquatain® Mosquito Formulation (AMF), for the control of the Afrotropical malaria vectors, *Anopheles gambiae sensu stricto* and *Anopheles arabiensis* in laboratory dose–response assays and standardized field tests.

**Methods:**

Tests were carried out following guidelines made by the World Health Organization Pesticide Evaluation Scheme (WHOPES). Sub-lethal effects of AMF were evaluated by measuring egg-laying and hatching of eggs laid by female *An. gambiae s.s.* that emerged from habitats treated with a dose that resulted in 50% larval mortality in laboratory tests.

**Results:**

Both vector species were highly susceptible to AMF. The estimated lethal doses to cause complete larval mortality in dose–response tests in the laboratory were 1.23 (95% confidence interval (CI) 0.99-1.59) ml/m^2^ for *An. gambiae s.s.* and 1.35 (95% CI 1.09-1.75) ml/m^2^ for *An. arabiensis*. Standardized field tests showed that a single dose of AMF at 1 ml/m^2^ inhibited emergence by 85% (95% CI 82-88%) for six weeks. Females exposed as larvae to a sub-lethal dose of AMF were 2.2 times less likely (Odds ratio (OR) 0.45, 95% CI 0.26-0.78) to lay eggs compared to those from untreated ponds. However, exposure to sub-lethal doses neither affected the number of eggs laid by females nor the proportion hatching.

**Conclusion:**

AMF provided high levels of larval control for a minimum of six weeks, with sub-lethal doses reducing the ability of female mosquitoes to lay eggs. The application of AMF provides a promising novel strategy for larval control interventions against malaria vectors in Africa. Further field studies in different eco-epidemiological settings are justified to determine the persistence of AMF film for mosquito vector control and its potential for inclusion in integrated vector management programmes.

## Background

Historically, larval source management made a significant contribution to many successful malaria control programmes [1–5]. The application of petroleum-based oils to water bodies to prevent emergence of adults is one of the oldest anti-larval measures used for mosquito control [6–8]. These petroleum-based oils kill the aquatic stages of mosquitoes by two mechanisms: specific toxicity and suffocation [9, 10] and provide effective control for two weeks or more [11, 12]. However, a major limitation of petroleum-based oils was the formation of a thick and non-uniform film that often required the addition of oil-soluble surface active agents to ensure uniform spreading of the film [13, 14]. Additionally, there are concerns about the damaging environmental consequences of these oils on non-target aquatic organisms when applications are made at high doses [15, 16]. Monomolecular surface films (MMFs) that consist of non-ionic surfactants were developed as potential alternatives to petroleum-based oils for mosquito control [17, 18]. A unique feature of MMFs is that they spread spontaneously and rapidly over a water surface to form a uniform ultrathin film about one molecule in thickness – a monolayer [17, 18]. Importantly, the effective doses used for mosquito control can be reduced 70 times when petroleum-based oils are replaced by MMFs [17], which saves on shipment, storage and application costs. Unlike petroleum-based oils and other control agents, MMFs are not toxic to immature mosquitoes [19, 20]. Their mode of action is physical, rather than chemical, and they work by lowering the water surface tension that affects all stages of the mosquito life-cycle; it is ovicidal, larvicidal, pupicidal and adulticidal [17, 19]. The reduced surface tension wets and drowns eggs, suffocates larvae and pupae and kills emerging and ovipositing females by drowning [19, 21]. This is an advantage over conventional insecticides that are only effective against larvae [22] or pupae [23]. Importantly, the physical mode of action reduces the chance of mosquitoes developing resistance [24].

Lecithin monolayers were the first MMFs to be evaluated for mosquito control but were only effective for two days when used to control *Anopheles gambiae sensu lato* in Western Kenya [25]. Arosurf ®MSF and Agnique®MMF are two commercially available MMFs made from renewable plant oils that are effective at controlling mosquitoes for up to five weeks in a variety of habitat types [18, 26–28]. However, MMFs are yet to gain wider acceptance in mosquito control programmes because of concerns about the disturbance of the film by environmental influences such as wind, rainfall and vegetation cover resulting in a patchy distribution of the chemical and mosquito emergence [18, 21, 29].

Aquatain® Mosquito Formulation (AMF) is a silicone-based film with a unique self-spreading ability. AMF was initially developed as an anti-evaporant to prevent water loss from large water reservoirs [24]. The advantage of the AMF film is its resilience to breakages by wind and rainfall as well as its ability to penetrate vegetation cover and floating debris on the water surface [24]. These properties combined with its safety to humans [24] make it a promising agent for mosquito control especially in large and highly vegetated habitats that have often proven difficult to treat with insecticides [30]. Surprisingly, to date only two studies have been published evaluating the potential of AMF for the control of *An. gambiae s.l*., the major malaria vector in sub-Saharan Africa; one laboratory [31] and one field [30] study.

We aimed to supplement the available knowledge by testing the efficacy of AMF for the control of *An. gambiae sensu stricto* and *An. arabiensis* in Phase I and Phase II trials following the standardized procedures by the World Health Organization Pesticide Evaluation Scheme (WHOPES) [32]. The specific aims of the study were to: (1) determine and compare the susceptibility of *An. gambiae s.s.* and *An. arabiensis;* (2) establish the initial and residual activity of AMF under standardized field conditions; and (3) test delayed effects of exposure to sub-lethal doses of AMF during larval development on a female’s ability to lay eggs, the number of eggs laid and the number of eggs hatched.

## Methods

### Study area

The study was carried out at the International Centre of Insect Physiology and Ecology, Thomas Odhiambo Campus (icipe-TOC) located on the shore of Lake Victoria in Homabay county, western Kenya (geographic coordinates 0° 26’ 06.19” S, 34° 12’ 53.13”E; altitude 1,137 m above sea level). The area is characterized by two rainy seasons, the long rains between March and June and the short rains between October and December. The average annual rainfall for 2010 to 2013 was 1, 645 mm (icipe-TOC meteorological station).

### Mosquitoes

Insectary-reared third instar larvae of *An. gambiae s.s.* and *An. arabiensis* (Mbita strains) were used for all experiments in this study. The mosquito immature stages were maintained in a netting-screened greenhouse-like building (semi-field system; 7.1 m wide, 11.4 m long and 2.8 m high at the wall and 4.0 m high at the highest point of the roof) [33] with an average daily temperature of 25-28°C, relative humidity of 68-75% and natural lighting. Mosquito maintenance is described more fully elsewhere [34]. Briefly, mosquito larvae were reared in round plastic tubs (diameter 60 cm) filled with 5 L water (5 cm deep) from Lake Victoria filtered through a charcoal-sand filter. The mosquito larvae were fed with fish food (Tetramin©Baby) twice daily. Mosquito larvae for experiments were randomly collected from different tubs to ensure that larvae introduced into each experimental cup or pond were of equal size [35].

### Insecticide

AMF was provided by the manufacturer Aquatain Products Pty Ltd., Australia. AMF contains 78% polydimethylsiloxane (silicone), the active ingredient. The manufacturer’s recommended application rate for mosquito control is 1 ml/m^2^.

### Dose–response tests

Tests were carried out on tables located in a semi-field system under ambient climatic conditions but protected from rain [33]. In range-finding tests, mortality rates were evaluated at doses between 0.01-1 ml/m^2^ compared to untreated controls. Thereafter, dose–response tests were carried out with dosages that yielded between 10% and 95% larval mortality in the range finding tests to determine the lethal doses, LD_50_, LD_90_ and LD_99_. Thus, the following dosages were evaluated: 0.05 ml/m^2^, 0.1 ml/m^2^, 0.2 ml/m^2^, 0.4 ml/m^2^ and 0.5 ml/m^2^. These were compared to larval mortality in untreated controls.

To carry out the tests, batches of 25 third-instar larvae were introduced into plastic tubs (diameter 0.42 m) filled with 5 L (depth 5 cm) of unchlorinated tap water originating from Lake Victoria. Thereafter, the appropriate volume of AMF was applied into the treatment tubs to obtain the above doses. Application of AMF was done using a micropipette. *Anopheles gambiae s.s.* and *An. arabiensis* were evaluated in parallel. The tests were conducted over three rounds on separate dates. Each test round lasted for 48 hours. Data on number of dead larvae was collected every 24 hours. Test larvae were fed on Tetramin©Baby fish food every 24 hours. In each round there were four replicates per test dosage and control for each mosquito species. Thus in total for each mosquito species there were 12 replicates per test dosage and control.

### Standardized field tests

**Figure 1:**
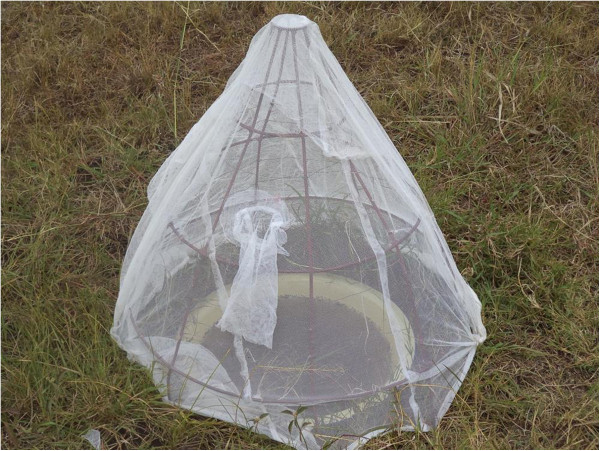
**Standardized field test set up.** Netting-covered emergence trap on top of artificial pond.

Tests were carried out in an open sunlit area within icipe-TOC campus that had been cleared of vegetation. Artificial ponds were created by sinking 40 plastic tubs, (diameter 0.42 m, depth 10 cm) into the ground. Ponds were arranged 1.5 m apart in eight rows with each row having five ponds. Each plastic tub was filled with 8 L of unchlorinated water and 2 L of soil to provide suitable biotic and abiotic parameters for mosquito larvae. Artificial ponds were used because tests were implemented during the dry season when natural breeding habitats of *An. gambiae* are often limited in number [36–38]. These tests were also conducted with insectary-reared *An. gambiae s.s.* and *An. arabiensis* larvae due to the low density of vectors in the study area during the dry season [39]. Both species were tested in parallel. Batches of 50 third-instar larvae were introduced into each pond before AMF was applied into treatment ponds; 20 ponds contained *An. gambiae s.s.* and 20 ponds contained *An. arabiensis.* The ponds were assigned into treatments and controls by lottery. Twenty ponds (10 per species) were treated with AMF at the manufacturer’s recommended dose of 1 ml/m^2^ [24]. Since the surface area of water in each pond was 0.14 m^2^, a volume of 0.14 ml (140 μl) was applied at the edge of the pond using a micropipette. The remaining 20 ponds (10 per species) were left untreated and served as controls. After AMF application an emergence trap modified from Fillinger *et al.* [40] was placed on top of each pond to prevent adult mosquitoes escaping and to avoid natural colonization of ponds by wild mosquitoes. A cone-shaped frame made of metallic rods was covered by mosquito netting with a sleeve to allow aspiration of any emerged adults (Figure 1).

The residual effect of a single dose of AMF was evaluated for six weeks by introducing new batches of 50 insectary-reared third-instar larvae into each pond each week. New batches of mosquito larvae were introduced into a pond using a plastic disposable transfer pipette (Fisherbrand, capacity 3 ml). This was done by first inserting the mouth of the pipette into the water before releasing the mosquito larvae gently into the water. After one week all larvae had developed into adults or died. After introducing larvae into each pond the number of live larvae and pupae and emerged adults was recorded daily. This was done by first assessing the emergence trap on each pond for presence of any emerged adult. If any adult was found in the trap it was aspirated into a holding plastic cup with the opening covered with mosquito netting. Emerged adults from separate ponds were held in separate holding plastic cups. At the end of a round, after six weeks, water from the ponds was discarded and set-up afresh for the next treatment round. The tests were conducted in three rounds. Rainfall was recorded at the icipe-TOC meteorological station weekly.

### Delayed effects in adults emerging from sub-lethal dosages

Forty artificial ponds (diameter 0.42 m) were set-up as described above in a semi-field system. Here the ponds were arranged in four parallel rows with 10 ponds in each row. Batches of 50 insectary-reared third instar *An. gambiae s.s.* larvae were introduced into each pond. Thereafter, 20 of the ponds were randomly selected and treated with AMF at 0.12 ml/m^2^, the dose that killed 50% of larvae in laboratory dose–response tests. To obtain this dose, 16.8 μl of AMF was applied at the edge of each treatment pond using a micropipette. The remaining 20 ponds were left untreated to serve as controls. Adult emergence from ponds was monitored as described above. The number of days to pupation was recorded. In addition the behaviour and movement of the larvae in water was observed. Tests were carried out in three rounds on separate dates with each round running for one week, sufficient for all larvae to successfully develop into adults or die. Every week, ponds were discarded to set-up the next treatment round with fresh batches of larvae.

Male and female mosquitoes that emerged from ponds were brought to the laboratory and transferred into 30 × 30 × 30 cm cages provided with 6% glucose solution *ad libitum*. Adults collected from control and treatment ponds were maintained in separate cages. Females in the cages were provided with a blood meal on a human arm on two consecutive days when they were 3–5 days old. On the third day after the last blood meal, gravid females were individually introduced into 15 × 15 × 15 cm cages that contained a glass cup (diameter 7 cm) filled with 100 ml unchlorinated tap water to serve as oviposition substrate. Mosquitoes were left overnight to lay eggs and the number of eggs laid by individual females the following morning was recorded. Eggs were left in the oviposition cups for three days to hatch. The number of eggs that hatched into larvae was recorded. Here the egg-laying capacity and hatching of eggs laid by 50 individual females collected from control ponds and 50 females from treatment ponds was evaluated in each round. Thus in total 150 individual females from control and 150 females from treated ponds were used in this test.

### Statistical analysis

IBM SPSS Statistics 20 software was used for data analyses. Dose–response data were analyzed using log-dosage probit regression analysis. All replicates of the dose–response tests were pooled by doses for each mosquito species to estimate the lethal dose that killed 50% of the population (LD_50_) and the LD_90_ and LD_99_. Test dosages were included in the model as covariates and mosquito species as factors. Relative median potency estimates were used to compare the susceptibility of mosquito species. Generalized estimating equations (GEE) fitted to a negative binomial distribution with a log-link function and an exchangeable correlation matrix were used to estimate the impact of treatment of ponds on adult emergence. The pond identity number was included as the repeated measure variable since data on larval mortality was repeatedly collected from the same pond. Treatment, mosquito species, application round, water turbidity (categorized as clear or turbid) and presence or absence of rain during the test week were included in the model as fixed factors. Interactions between treatment and turbidity, and treatment and rain were also included in the model. A GEE model was also used to estimate the delayed effect of exposure of *An. gambiae s.s.* to sub-lethal dosages in the larval habitat on egg-laying and hatching of eggs. The parameter estimates of the GEE models were used to predict the weekly mean adult emergence, mean number of eggs laid per female and mean number of eggs that hatched into larvae and their associated 95% confidence intervals by removing the intercept from the models. Weekly percent reductions in adult emergence from treated ponds was calculated with Abbott’s formula [41]. The time to pupation of larvae introduced into ponds in tests to evaluate sub-lethal effects of AMF was calculated using the formula: (Ax1) + (Bx2) + (Cx3)^**….**^ + (Hx8)/(Total number of pupae collected) where A, B, C^**…..**^H are the number of pupae collected on day 1, 2, 3 to 8.

### Ethical considerations

Ethical approval for arm-feeding mosquitoes was obtained from the Kenya Medical Research Institute’s Ethical Review Committee. An experimental permit to import and test AMF was granted by the Pest Control Products Board, Nairobi, Kenya.

## Results

### Dose–response tests

**Figure 2:**
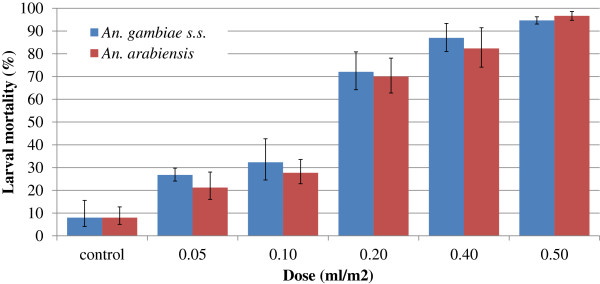
**Mean mortality of larvae exposed to increasing doses of AMF in dose–response tests.** Error bars represent 95% confidence intervals.

Larval mortality was similar in the three experimental rounds for each mosquito species; therefore rounds were pooled for each mosquito species for calculation of mean larval mortality and effective lethal doses. The relative median potency estimates showed that both mosquito species were equally susceptible to AMF. Larval mortality occurred at all doses tested (Figure 2). Probit analysis predicted that approximately 0.5 ml/m^2^ was required to kill 90% of all exposed larvae whilst slightly over 1 ml/m^2^ of AMF was needed to kill all larvae after 48 hours of exposure (Table 1). It was observed that at the two lower doses of AMF, 0.05 and 0.1 ml/m^2^, some parts of the water surface remained untreated. Observation of the larvae in tubs treated at dosages above 0.1 ml/m^2^ showed a reduced activity compared to larvae in control tubs and very slow response rates when disturbed e.g. when passing a hand over water surface or tapping the larval container. Larvae exposed to higher doses of AMF were often observed to coil into a circle with their mouthparts placed on the abdomen in a tail nibbling effect.

**Table 1:** **Effective doses of AMF against third instar** ***An. gambiae s.s.*** **and** ***An. arabiensis***

	***Anopheles gambiae s.s.***	***Anopheles arabiensis***
	ml/m^2^	ml/m^2^
LC_50_ (95% CI)	0.12 (0.11-0.13)	0.13 (0.11-0.15)
LC_90_ (95% CI)	0.43 (0.37-0.51)	0.47 (0.41-0.56)
LC_99_ (95%CI)	1.23 (0.99-1.59)	1.35 (1.09-1.76)

### Standardized field tests

**Figure 3:**
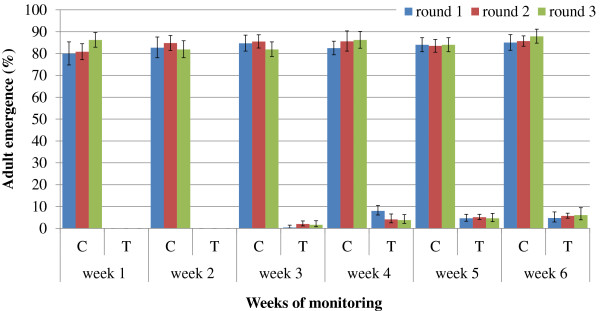
**Weekly emergence of** ***An. gambiae s.l.*** **from control (C) and treatment (T) in standardized-field tests.** Error bars represent 95% confidence intervals.

The effect of AMF on larval mortality under field conditions was not significantly different between *An. gambiae s.s.* and *An. arabiensis* (Table 2) thus data for the two species were pooled to show weekly larval mortality in Figure 3 and to calculate weekly percent mortality (Table 3). AMF applied at 1 ml/m^2^ provided complete larval mortality for two weeks. Emergence from treatment ponds occurred from week 3, but this remained below 10% over the six week monitoring period (Figure 3). The emergence of adults coincided with the observation of small breakages of the surface film in some of the ponds from the third week onwards. On average, 84.7% (95% 75.7-93.3%) of larvae introduced weekly into control (untreated) ponds successfully developed into adults. Results were very consistent from round to round (Figure 3).

**Table 2:** **GEE analysis of factors affecting adult emergence from ponds**

Explanatory variables	Odds ratio (95% CI)	p-value
**Treatment**		
Treatment ponds	0.15 (0.12-0.18)	<0.0001
Control ponds	1	
**Mosquito species**		
*An. gambiae s.s.*	0.94 (0.85-1.04)	0.235
*An. arabiensis*	1	
**Round**		
Round 3	1.05 (0.93-1.19)	0.408
Round 2	1.09 (0.95-1.24)	0.223
Round 1	1	
**Weeks**		
Week 6	2.61 (1.70-4.02)	<0.0001
Week 5	2.37 (1.60-3.51)	<0.0001
Week 4	2.71 (1.78-4.10)	<0.0001
Week 3	1.35 (1.12-1.64)	0.002
Week 2	1.01 (0.93-1.10)	0.778
Week 1	1	
**Water turbidity**		
Turbid	0.65 (0.51-0.82)	<0.0001
Clear	1	
**Rainfall**		
Rain	0.80 (0.68-0.95)	0.013
No rain	1	
**Interaction between treatment and turbidity**		
Treatment*turbid	2.72 (1.99-3.72)	<0.0001
Treatment*clear	1	
**Interaction between treatment and rainfall**		
Treatment*rain	1.45 (0.95-2.11)	0.053
Treatment*no rain	1	

*symbol for interaction between factors.

**Table 3:** **Weekly percent mortality of** ***An. gambiae s.l.*** **larvae in treatment ponds**

	Week 1	Week2	Week 3	Week 4	Week 5	Week 6
Round 1	100	100	97 (96–99)	90 (87–92)	94 (92–96)	94 (91–96)
Round 2	100	100	97 (96–98)	95 (92–97)	93 (92–95)	93 (92–94)
Round 3	100	100	95 (94–99)	95 (93–97)	94 (92–96)	93 (90–95)

Adjusting for other factors, it was 6.7 times less likely for an adult to emerge from treated ponds compared to control ponds (Table 2). However, the probability of emergence increased over time and was 1.4-2.6 times higher from ponds that had received treatment 3–6 weeks earlier compared to freshly treated ponds (Table 2). Both turbidity and rainfall affected adult emergence from ponds irrespective of the treatment. It was 1.5 times less likely for adults to emerge from turbid ponds than from clear ponds and 1.25 times less likely to emerge if it had rained during the exposure week (Table 2). In addition to the main effect, turbidity and rainfall interacted with the treatment in such a way that both factors increased the probability of emergence from AMF treated ponds, or in other words, slightly decreased the impact of the intervention (Table 2). The overall impact of the interaction can be estimated by multiplying the odds ratios [42]. This means for instance that while it was 6.7 times less likely for adults to emerge from treated ponds that were clear in the first week of round 1, it was only 3.8 times less likely for adults to emerge from treated ponds that were turbid in the same time period. Similarly, while it was 4.5 times less likely for adults to emerge from treatment ponds when it failed to rain during week 3 of round 2, the likelihood of emergence was only 3.8 times less from similar treatment ponds at same time period when it rained.

### Delayed effects in adults emerging from sub-lethal dosages

Results from individual rounds were similar (p = 0.16) and therefore pooled for analysis. The mean percent adult emergence was 92.9% (95% CI 92.4-93.3%) from untreated ponds and 55.8% (95% CI 44.9-66.5%) from treated ponds. Significant differences were observed in the mean pupation time of larvae introduced in control and treatment ponds. Of those larvae that survived, the mean pupation time was estimated as 3.4 days (95% CI 3.0-3.7) in control ponds and 4.9 days (95% CI 4.4-5.3) in ponds treated with sub-lethal dose of AMF. Furthermore, live larvae in treated ponds often showed signs of weakness as they exhibited slow movement when disturbed on the water surface in contrast to those unexposed.

Females that emerged from ponds treated with sub-lethal doses of AMF were 2.2 times less likely (OR 0.45; 95% CI 0.26-0.78) to lay eggs compared with females from untreated ponds. However, if females laid eggs the mean number of eggs laid per female did not differ significantly between treatment groups (p = 0.31). The mean number of eggs laid per female was 49.3 (95% CI 41.3-58.8) when adults emerged from control ponds and 45.4 (95% CI 37.4-55.1) when females emerged from larvae that developed in ponds treated with a sub-lethal dose of AMF. Similarly, there were no significant differences in the hatching of eggs laid by females emerged from treated and control ponds (p = 0.18). The mean number of hatched eggs was 41.0 (95% CI 38.0-44.2) when eggs were laid by females emerging from control ponds and 36.8 (95% CI 33.8-40.1) for eggs laid by females emerging from ponds treated with a sub-lethal dose of AMF.

## Discussion

The dose–response tests and consequent standardized field tests confirmed that the manufacturer’s recommended dosage of 1 ml/m^2^ is effective for the control of the two malaria vectors, *An. gambiae s.s.* and *An. arabiensis*. Furthermore, the dose–response tests highlight the high susceptibility of these two species with half the recommended dosage (0.5 ml/m^2^) already leading to 90% mortality and approximately a quarter of it still leading to greater than 50% mortality. *Anopheles gambiae s.s.* and *An. arabiensis* were equally susceptible to AMF which is not surprising given the physical mode of action of this larvicide and the similar larval behaviour of both vector species [43] exposing them to the surface film while feeding.

The standardized field tests showed over 80% emergence inhibition from AMF-treated ponds over the entire six week observation period, confirming the stability of the silicone-based surface film over time. Our results confirm the extended residual activity of AMF and other MMFs reported in the field [18, 26, 44]. Studies have shown that Arosurf® MSF and Agnique® MMF are effective for control of different genera of mosquito for 7–21 days [18, 28]. The efficacy of AMF was found to last 4–6 weeks for the control of *Culex* and *Aedes* larvae in small-scale field trials in Australia [44]. It is important, however, to consider that our test habitats were small, confined and undisturbed and phase III trials should now be conducted to evaluate AMF in different habitat types and sizes to establish the residual activity under different environmental conditions to give final recommendations for application intervals for different habitat types. The only field study to evaluate AMF for control of Afrotropical malaria vectors found the film to be effective in reducing emergence of anopheline and culicine mosquitoes when applied at 1 ml/m^2^ in rice paddies in Western Kenya [30]. However, a double dose (2 ml/m^2^) was necessary to effectively suppress larval densities of both mosquito genera [30]. Differences in susceptibility of life stages of mosquito immatures to surface films have been reported elsewhere [18, 31].

Turbid water and rainfall reduced the efficacy of AMF for mosquito control. The water in our artificial ponds could have been turbid due to algae, bacteria and other suspended particles in the water column [45]. Possibly turbidity increased the rate of degradation of the AMF film therefore reducing film efficacy from the effect of increased water temperatures [29, 46–48]. It might also be that the reduced efficacy of the film in turbid water is caused by natural films formed by suspended particles that limit the spread of AMF film [17]. Rainfall in general increased larval mortality irrespective of the treatment likely due to flush out effects [49]. However, larvae from treated ponds that experienced rain during the week of exposure were more likely to survive than larvae from treated ponds without rain, probably because rain breaks up the surface film and provides pockets of film free environments for larval development [29]. It has been reported in other studies that rainfall is a major factor that limits the efficacy of surface films for mosquito control [21, 29], though in our study rainfall reduced the activity of AMF only slightly. However, this tool would be especially promising when applied to aquatic habitats in the dry season due to the minimal climatic and environmental influences at this time providing long-lasting control with a single application.

Exposure of larval stages to sub-lethal doses of AMF increased larval development time and reduced the proportion of gravid females egg-laying. Similar effects have been reported for organophosphates, spinosyns, insect growth regulators and microbials [50–56]. These effects would be an additional benefit to larviciding programmes as they reduce the frequency of larvicide application thereby reducing intervention costs [57]. Longer larval development time predisposes mosquito larvae to several risks that reduce their survival including predation, disturbances by human activities and instability of breeding habitats [58–60]. It has been previously shown that nutrient deprivation is a common cause of prolonged mosquito larval development [58, 61–64]. Thus, it is most likely in the current study the prolonged larval development was caused by poor nutrition of larvae in treatment ponds. This is because as observed in our dose–response tests and previous studies [19, 65], mosquito larvae exposed to MMFs spend a great deal of time attempting to wash off the liquid that blocks their respiratory structures and thus have little time to feed. Adults that emerge from poorly fed larvae are often small in size with low teneral reserves [58, 62, 66], with the effect of reduced egg-laying capacity [67, 68], a phenomenon observed in the current study. Additional effects of reduced survival and insemination in females have been observed in adults deprived of nutrients during the larval stage [58, 69], which can potentially reduce the vectorial capacity.

## Conclusion

The high susceptibility of *An. gambiae s.s.* and *An. arabiensis*, the long residual activity, sub-lethal effects on larval development and reproduction combined with the physical mode of action makes AMF a novel, and potentially important tool for larval control interventions against malaria vectors in Africa. Further field studies in different eco-epidemiological settings are justified to determine the efficacy and persistence of AMF film for mosquito vector control and its potential for inclusion in integrated vector management programs. Furthermore, although AMF and other MMFs have been shown to have minimal effect on most non-target aquatic insects since they spend much less time on the water surface [18, 28, 30], concerns on the safety of those that rely on the water surface for respiration and movement needs to be investigated. AMF might be a useful control agent to be considered for rotation or in combination with other larvicides to reduce insecticide-resistance development.
